# Case Report: Rapid and Effective Treatment of Porokeratosis With Fractional CO_2_ Laser Combined With Topical 2% Cholesterol/Lovastatin Ointment

**DOI:** 10.1111/jocd.70289

**Published:** 2025-06-13

**Authors:** Pingping Gao, Xiaolong Zhang, Haoyu Yang, Jing Wang

**Affiliations:** ^1^ Department of Dermatology The Second Affiliated Hospital of Anhui Medical University Hefei Anhui China

**Keywords:** cholesterol, disseminated superficial actinic porokeratosis, fractional CO_2_ laser, lovastatin

To the Editor,

## 1 Introduction

Disseminated superficial actinic porokeratosis (DSAP) is a group of autosomal dominantly inherited keratinization disorders of the epidermis, which is one of the major subtypes of porokeratosis (PK). Although the first case was reported more than a century ago, its etiology and pathogenesis are not fully understood. Liquid nitrogen cryotherapy, skin grinding, podophyllotoxin, retinoids, and other treatments have been reported to treat perspiration keratosis, but they are not very effective or have recurrent symptoms. Herein, we report a case of DSAP that was successfully treated with fractional CO_2_ laser combined with 2% topical cholesterol/lovastatin ointment.

## 2 Case Report

**FIGURE 1:**
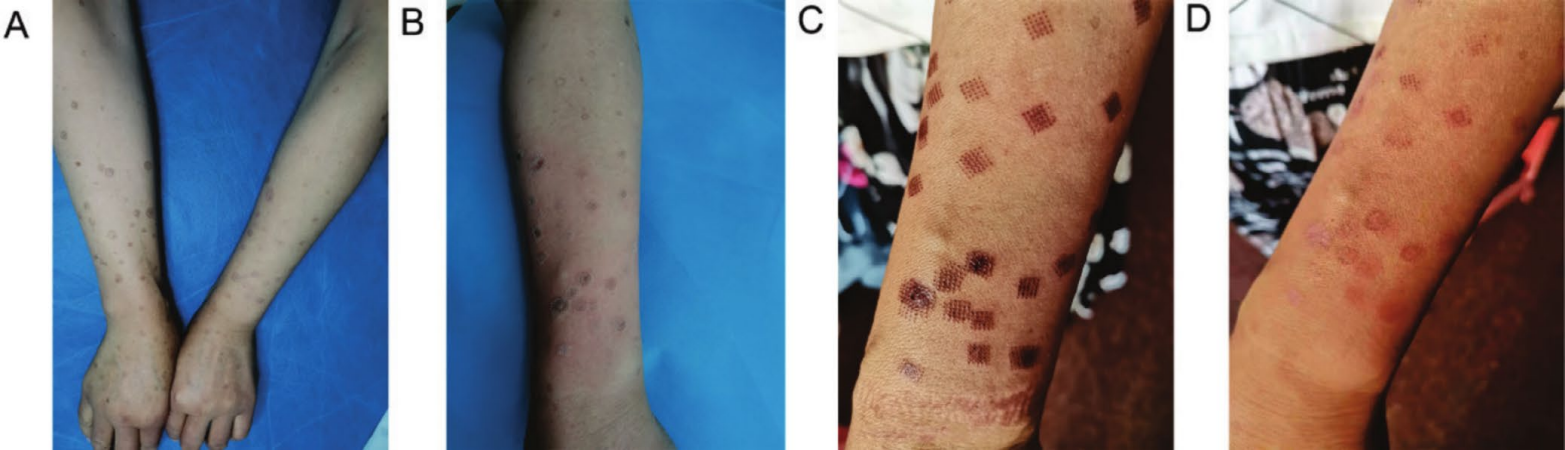
Clinical appearance of the left forearm: (A) before the treatment, (B) immediately after fractional CO_2_ laser treatment, (C) 1 week after treatment, and (D) 2 weeks after treatment.

**FIGURE 2:**
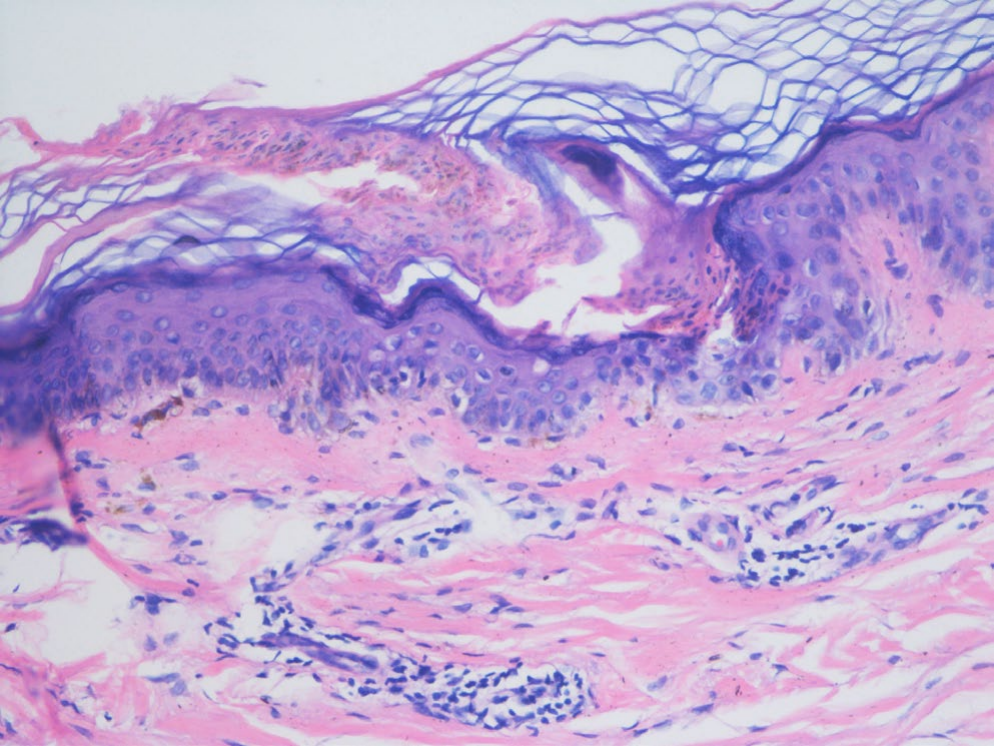
There were cornoid lamellae in the epidermis and no granular cell layer (H&E × 200).

A woman visited the Dermatology Department of the Hospital, with skin lesions that had initially manifested in her twenties. The patient's son had similar lesions. The patient did not have any history of systemic diseases or underlying conditions. Results of the patient's laboratory tests, including blood cell count, liver function, kidney function, fasting blood glucose, blood lipids, erythrocyte sedimentation rate, C‐reactive protein, HIV, and hepatitis B and C, were normal. Physical examination showed many uneven annular lesions distributed on the sun‐exposed skin areas, such as the face, forearms, hands, and neck (Figure 1A). The lesions had variable color and were surrounded by raised keratinized ridges. Histopathological examination revealed cornoid lamella in the epidermis and no granular cell layer (Figure 2). Based on the clinical symptoms and histopathological examination, the patient was diagnosed with DSAP.

With the patient's consent, we selected the skin lesions on the patient's forearm for treatment. To increase the absorption of the drug, we first treated the skin lesions with fractional CO_2_ laser (CO2RE, Syneron Candela), (10 600 nm wavelength, Deep Mode, 50 mj energy, 5% coverage) and then applied topical 2% cholesterol/lovastatin ointment twice a day. Laser treatment is only once, and topical cream is used twice daily for 2 weeks. In the subsequent follow‐up visit, we observed that some lesions had disappeared, the color of the lesion center became lighter, and the keratinized ridges disappeared after the laser scab dislodged (Figure 1B–D). In addition, the patient showed no recurrence of the lesions in the 6‐month follow‐up.

## 3 Discussion

DSAP is the most common type of porokeratosis, which occurs mostly at the exposed site and worsens in the summer. The main features are superficial, small, clustered, numerous skin lesions in the limbs. The early lesions are small keratinized papules with a central fovea. After gradually expanding, the skin lesions subsequently become superficial and circular, with slight atrophy in the center and a dike‐shaped uplift. There is currently no specific treatment for the disease, and the available oral and topical retinoids, topical 5‐fluorouracil cream, cryotherapy, laser, skin grinding, and other treatments are not very efficacious. Cholesterol is one of the end products of the mevalonate pathway and a major component of the skin barrier. Cholesterol deficiency can lead to premature apoptosis of keratinocytes, which in turn leads to the porokeratosis phenotype. Some studies have shown that porokeratosis might be similar to other metabolic diseases, loss‐of‐function mutations, resulting in excessive toxic intermediate metabolites and/or insufficient end products [1]. Therefore, reducing the accumulation of intermediate metabolites and supplementing downstream products may be helpful for the treatment of porokeratosis. Atzmony et al. [2] showed that cholesterol/lovastatin compound could reduce symptoms of porokeratosis in a patient with DSAP. Blue et al. [3] reported a patient with linear porokeratosis and bone abnormalities who was successfully treated with topical 2% cholesterol/lovastatin ointment. Maronese [4] also found that topical cholesterol/lovastatin is a safe and effective therapy for porokeratosis. This case report used fractional CO_2_ laser combined with topical 2% cholesterol/lovastatin ointment, which were observed to have a curative effect in 2 weeks, and no recurrence in 6 months of follow‐up, was definitely better than single laser therapy or topical drug treatment.

## 4 Conclusion

This case report increases the number of reported PK cases successfully treated with topical 2% cholesterol/lovastatin ointment. Although some previous reports have shown that topical 2% cholesterol/lovastatin ointment can treat porokeratosis, fractional CO_2_ lasers are not used in combination. Fractional CO_2_ laser technology increased and accelerated the absorption of drugs, which resulted in rapid and successful treatment. To the best of our knowledge, this is the first case of DSAP that was successfully treated with fractional CO_2_ laser, which enhanced transdermal absorption of 2% compound cholesterol/lovastatin ointment. However, this method needs further verification with a large sample size.

## Author Contributions

Jing Wang conceived and supervised the study; Pingping Gao, Xiaolong Zhang, and Haoyu Yang analyzed data; Pingping Gao wrote the manuscript; Jing Wang made manuscript revisions. All authors reviewed the results and approved the final version of the manuscript.

## Conflicts of Interest

The authors declare no conflicts of interest.

## Data Availability

The authors confirm that the data supporting the findings of this study are available within the article.
